# Correction: Gamma ray assisted modification of carbon quantum dot/polyurethane nanocomposites: structural, mechanical and photocatalytic study

**DOI:** 10.1039/d0ra90015j

**Published:** 2020-02-17

**Authors:** Milica Budimir, Zoran Marković, Dragana Jovanović, Miloš Vujisić, Matej Mičušík, Martin Danko, Angela Kleinová, Helena Švajdlenková, Zdeno Špitalský, Biljana Todorović Marković

**Affiliations:** School of Electrical Engineering, University of Belgrade Bulevar kralja Aleksandra 73 11000 Belgrade Serbia budimir@vinca.rs; Vinča Institute of Nuclear Sciences, University of Belgrade Mike Petrovića Alasa 12-14 11001 Belgrade Serbia; Polymer Institute, Slovak Academy of Sciences Dúbravská cestá 9 84541 Bratislava Slovakia

## Abstract

Correction for ‘Gamma ray assisted modification of carbon quantum dot/polyurethane nanocomposites: structural, mechanical and photocatalytic study’ by Milica Budimir *et al.*, *RSC Adv.*, 2019, **9**, 6278–6286.

The authors regret that the primary affiliation of the corresponding author (Milica Budimir) was incorrectly designated in the original manuscript. The corrected list of affiliations is as shown herein.

The Royal Society of Chemistry apologises for these errors and any consequent inconvenience to authors and readers.

## Supplementary Material

